# A rare cardiac phenotype of dextrocardia observed in a fetus with 1p36 deletion syndrome and a balanced translocation: a prenatal case report

**DOI:** 10.1186/s13039-020-00514-1

**Published:** 2020-11-16

**Authors:** Li Gao, Junyu Zhang, Xu Han, Wenjing Hu, Jinling Sun, Yuru Tan, Xinrong Zhao, Renyi Hua, Shan Wang, Yan Zhang, Yanlin Wang, Yi Wu

**Affiliations:** 1 Prenatal Diagnostic Center, International Peace Maternity and Child Health Hospital, School of Medicine, Shanghai Jiao Tong University, Shanghai, China; 2 Department of Reproductive Genetics, International Peace Maternity and Child Health Hospital, School of Medicine, Shanghai Jiao Tong University, Shanghai, China; 3 Shanghai Key Laboratory of Embryo Original Disease, Shanghai, China

**Keywords:** 1p36 deletion syndrome, Prenatal diagnosis, Isolated dextrocardia, Chromosomal microarray analysis, Whole genome sequencing

## Abstract

**Background:**

Chromosome 1p36 deletion syndrome is a contiguous genetic disorder with multiple congenital anomalies and mental retardation. It has been emerging as one of the most common terminal deletion syndromes in humans with the rapid utility of microarray analysis. However, the prenatal findings of 1p36 deletion syndrome are still limited. We report a fetus with 1p36 deletion and cardiac phenotype of dextrocardia, combined with a balanced translocation between chromosome 5 and 6. The phenotype of dextrocardia is rarely reported in prenatal 1p36 deletion cases.

**Case presentation:**

We present a prenatal 1p36 deletion case with congenital heart diseases and single umbilical artery. Fetal echocardiography showed dextrocardia, ventricular septal defect and pericardial effusion. Fetal karyotype revealed a de novo balanced translocation of 46,XY,t(5;6)(q11.2;q23.3). Chromosomal microarray analysis detected a pathogenic deletion in 1p36.21p36.12, with the size of 6.38 Mb. Further whole genome sequencing revealed that the balanced translocation disrupted the *EYA4* and *ITGA1* genes.

**Conclusions:**

Although congenital heart diseases are very common clinical manifestations among patients with 1p36 deletion, dextrocardia is a quite rare cardiac phenotype. This is the second case with 1p36 deletion and dextrocardia, and the first prenatally diagnosed 1p36 deletion case with dextrocardia. Our case indicates that genes in 1p36 are associated with not only heart structural anomalies, but also cardiac laterality development. Our results also imply that the *EYA4* gene disrupted by the balanced translocation might be related with the cardiac development.

## Background

Chromosome 1p36 deletion syndrome was first described by Yunis et al. [1]. It is a contiguous gene syndrome with great variation in genetic deletions. The clinical problems caused by this syndrome include typical facial features, multiple congenital anomalies (especially brain and heart), neurodevelopmental delay, growth retardation, and other unspecific clinical manifestations such as hearing loss and vision problems, etc. [2, 3]. Its estimated incidence is approximately 1 in 5000–10,000 live births [4]. During the last decade, the incidence of this syndrome is increasingly higher due to the widespread use of microarray analysis. However, the prenatal features of 1p36 deletion syndrome are still limited. Here we report a fetus with 1p36 deletion and a very rare cardiac phenotype of dextrocardia.

## Case presentation and methods

A 28-year-old primigravid woman was referred to our center due to suspected fetal congenital heart disease. The couple is not consanguineous. The pregnancy was unremarkable until the second trimester. The fetal anomaly scan showed fetal biparietal diameter(BPD) 62 mm, head circumference (HC) 216 mm, and femur length (FL) 38 mm at 24 weeks. Single umbilical artery was observed and fetal congenital heart defect was suspected.

**Fig. 1:**
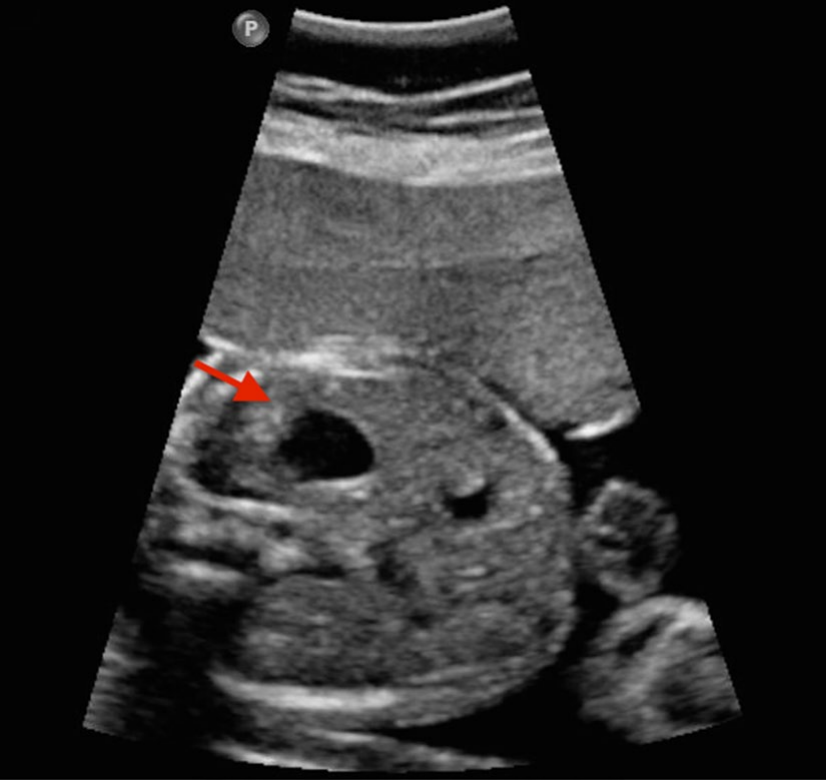
The red arrow is pointed to the stomach bubble, which was located in the left side

**Fig. 2:**
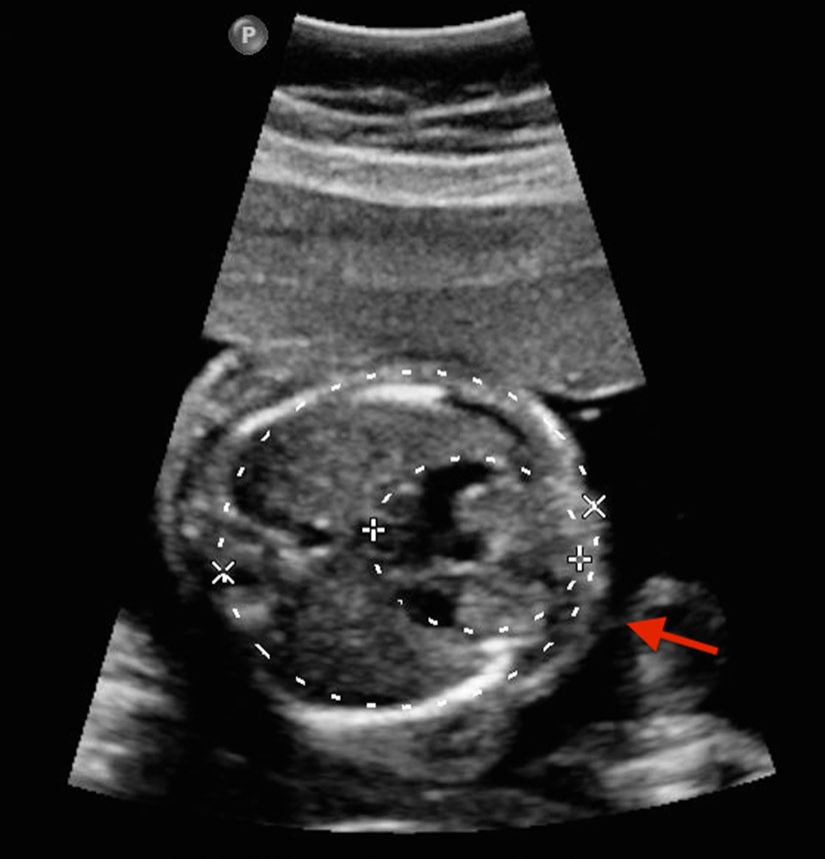
The apical part of the heart was to the right (seen in the red arrow), indicating the presence of dextrocardia

**Fig. 3:**
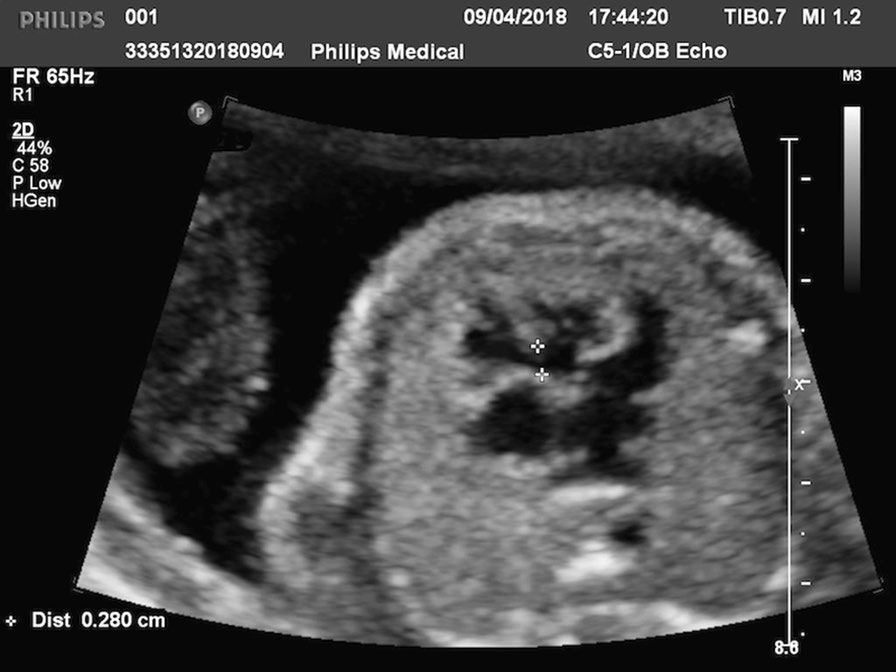
VSD

Fetal echocardiography was soon performed using Ge Voluson E10 multifunctional doppler ultrasound instrument and dextrocardia was observed (Figs. 1, 2). Fetal echocardiography also displayed ventricular septal defect (VSD) with the size of 2.8 mm (Fig. 3). The fetus also had a pericardial effusion with the diameter of 4.4 mm.

**Fig. 4:**
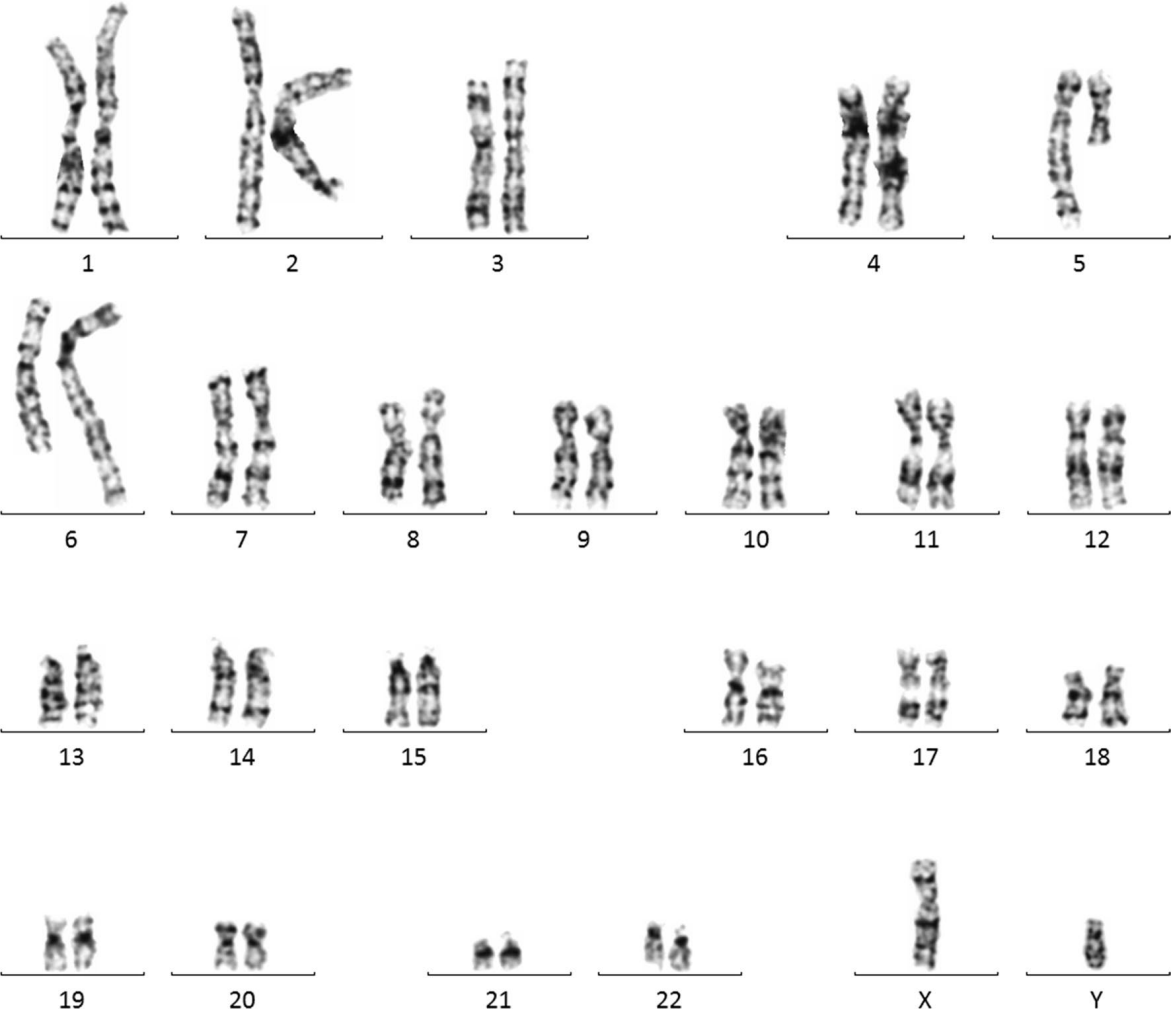
Fetal karyotype result

The amniocentesis was performed to obtain the fetal samples after signing the informed consent as described previously [5]. Amniotic fluid cells were cultured according to the standard protocol in two independent flasks. Chromosomal karyotyping was performed according the standard protocol using G-banding. Five metaphase cells were carefully examined by an experienced technician to detect structural chromosomal abnormalities, and at least 15 metaphase chromosomes were looked at to detect numerical abnormalities of chromosomes. Chromosomal karyotyping revealed a balanced translocation of 46,XY,t(5;6)(q11.2;q23.3) (Fig. 4). To investigate the inheritance, parental peripheral blood samples were collected and cultured. The maternal karyotype showed a pericentric inversion of chromosome 9,46,XX,inv(9)(p12q13),21pstk+, which was not considered to be associated with fetal 1p36 deletion. The paternal karyotype was normal.

**Fig. 5:**
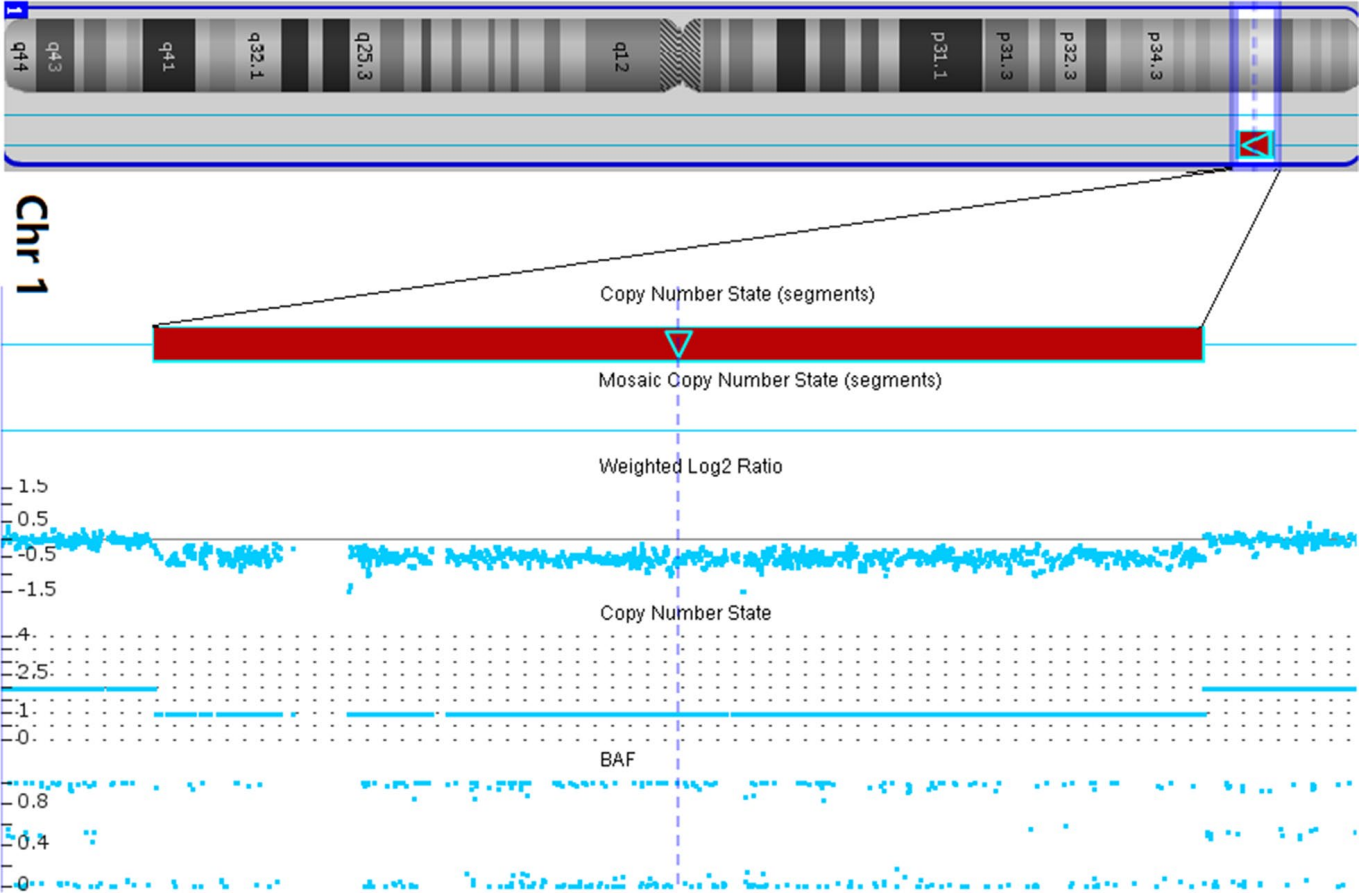
CMA result revealed a pathogenic deleiton of 1p36.21p36.12:arr[hg19] 1p36.21p36.12(16105084_22493485) × 1

Genomic DNA was extracted from 5 ml amniotic fluid using Tianamp Micro DNA Kit (Tiangen, Biotech, Beijing, China), according to manufacturer’s protocol. DNA was screened with Affymetrix Cytoscan 750 K (Affymetrix Santa Clara, Clifornia). Result was analyzed with Affymetrix Chromosome Analysis Suite software (ChAS).3.2(Affymetrix, Santa Clara, California). The CMA result showed a partial monosomy 1p, arr[hg19] 1p36.21p36.12(16105084_22493485) × 1 (Fig. 5). No abnormality was detected in both parental peripheral blood CMA, suggesting a de novo occurrence.

**Fig. 6:**
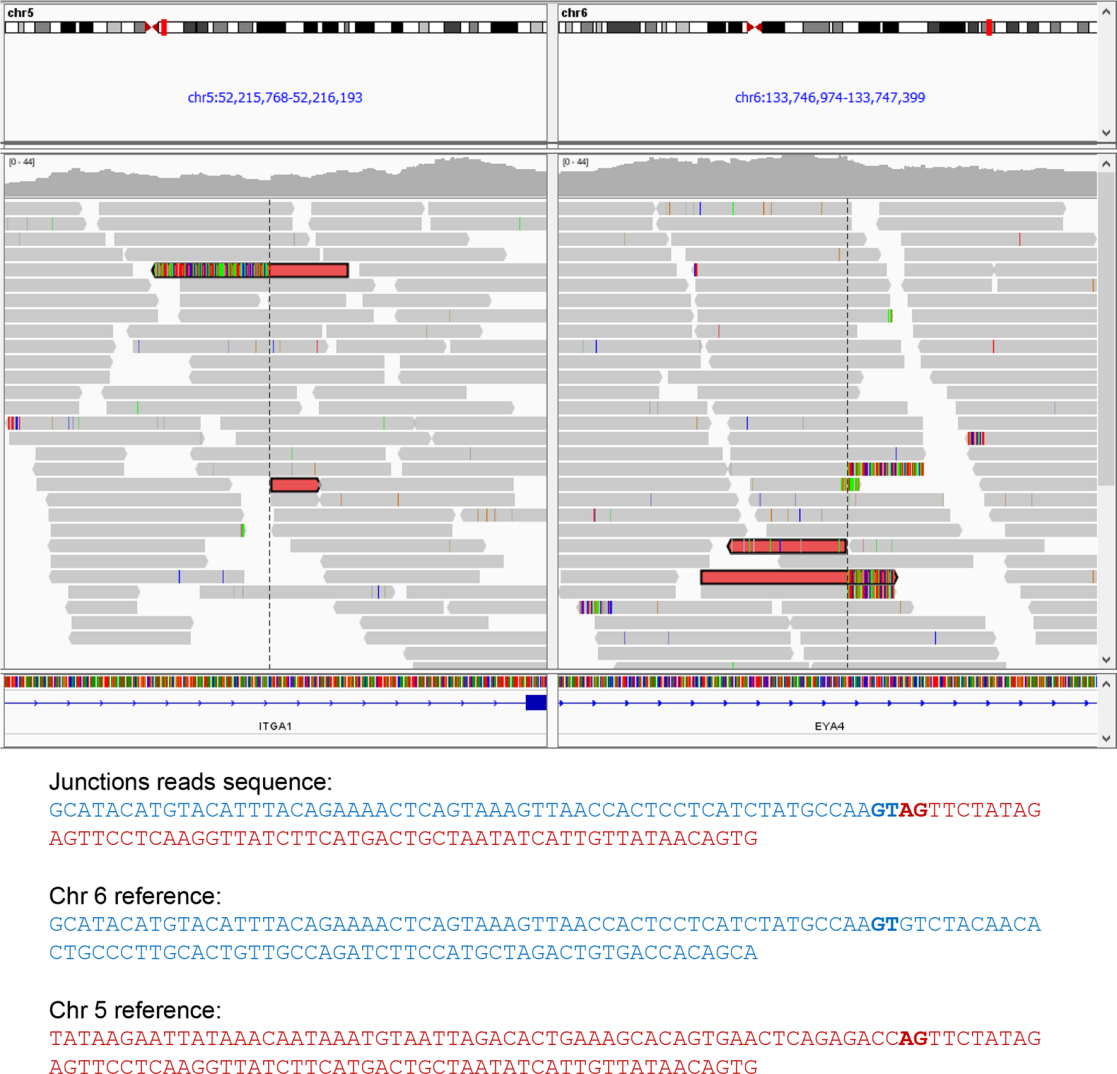
Integrative Genomics Viewer (IGV) screenshot of whole genome sequencing pair-end reads of the fetus. The dashed lines indicate the breakpoints on chromosomes 5 and 6. Junctions reads sequence are shown below

To investigate the potential genes disrupted in the translocation breakpoints, we performed whole genome sequencing (WGS) using the fetal DNA extracted from the amniotic fluid cells. The WGS was performed using HiSeq 2500 platform (Illumina) with 150-bp paired-end reads. The structural variant breakpoint analysis was performed as previously described [6].The structural variant breakpoint analysis revealed that the breakpoint on chromosome 6 located at NC_000006.11:g.133747301, which led to the disruption of the *EYA4* gene. The other breakpoint on chromosome 5 located at NC_000005.9:g.52215965, which lead to the disruption of *ITGA1* gene (Fig. 6) (The bam file is available upon request).

Finally, the parents decided to terminate the pregnancy at 26 weeks of gestation and declined fetal autopsy after genetic counselling.

## Discussion and conclusions

Chromosome 1p36 deletion syndrome is merging as the most common deletion syndrome with the rapid utility of microarray analysis. In postnatal cases, this syndrome is characterized by facial dysmorphism, structural abnormalities, mental retardation, developmental delay, etc. Cardiac abnormalities are very common clinical presentations among 1p36 deletion patients, including ventricular septal defect, patent ductus arteriosus, atrial septal defect, Ebstein anomaly, tetralogy of Fallot, etc. [7]. According to the literature review, approximately 50–70% affected postnatal individuals have cardiac structural anomalies. Compared with the postnatal reports, the fetal congenital heart disease (CHD) phenotypes associated with 1p36 deletion were still rare.

In the present study, our case showed CHD of an isolated dextrocardia without other viscera transposition. Isolated dextrocardia is a very rare CHD phenotype among 1p36 deletion patients. To the best of our knowledge, isolated dextrocardia has never been reported in prenatal 1p36 deletion cases. Even in postnatal individuals, isolated dextrocardia is extremely seldom observed except one 11-year-old male patient reported by Puvabanditsin et al. [8]. This male patient presented dextrocardia and ventricular septal defect, combined with a dysmorphic face and speech delay. The genetic microdeletion with the size of 4.8 Mb in this male patient located in 1p36.33, which did not overlap with that in our fetus. Zaveri et al. [9] proposed several critical regions for CHD in 1p36 region. The microdeletion seen in our case overlapped with one of the CHD critic regions in 1p36.21p36.12(12726755-23438888). The microdeletion seen in Puvabanditsin’s patient overlapped with another CHD critical region in 1p36.33(1-2418935) [8]. Both of two cases supply more insight that 1p36 deletion is not only associated with cardiac structural growth, but also fetal heart laterality development.

Several genes in the deleted interval of our case were found to be associated with cardiac structural malformations. The gene *SKI* (chr1:2160134-2241652; OMIM#164780) is one of them. Zhu et al. found a patient with atrial septal defect harbored a very small 576 kb microdeletion of 1p36.33-p36.32 only containing *SKI*, which indicates this gene might contribute to cardiac development [10]. *SKI* functions as a repressor of TGF-beta signaling, the latter is an important signaling pathway involving cardiac development [11]. Haploinsufficiency of *SKI* might lead to cardiac malformation through affecting the TGF-beta signaling pathway. Another candidate gene which might be involved in heart development located in the deleted interval is *SPEN*(chr1:16174359-16266950; OMIM #613484). Kuroda et al. found that Spen-null embryos die in utero and have defective formation of the cardiac spectrum, suggesting *SPEN* is the necessary gene in heart development [12]. However, up to now, no genes in 1p36 region have been reported to be associated with cardiac laterality development. Our study cast a new light on the association of 1p36 genes and cardiac laterality development. Further studies are warranted to confirm the conclusion and investigate the underlying molecular mechanisms.

Chromosome rearrangement is a very complex process. The types of rearrangements seen in 1p36 deletion cases include derivative unbalanced translocations and complex rearrangements such as interrupted inverted duplications or triplications [4]. Interestingly, the translocation seen in our fetus does not belong to any kind. In consideration of the de novo balanced translocation may contribute to the phenotypes of the fetus,translocation breakpoints were mapped using WGS. The breakpoint on chromosome 6 disrupted the *EYA4* gene, which is an HI gene with ClinGen HI score of 3. Truncating *EYA4* mutations cause late-onset autosomal dominant dilated cardiomyopathy. However, the patients with *EYA4* mutations were characterized by progressive and late-onset in reported cases [13, 14]. Thus, the relationship between the disruption of *EYA4* gene and CHD phenotypes in our case is still need to be further studied. The other breakpoint on chromosome 5 disrupted the *ITGA1* gene, which has not been associated with any genetic disease. Studies using animal model showed that the gene might be associated with cartilage development [15, 16]. Although no pathogenic variants explaining the phenotype-genotype correlation were detected, WGS provided valuable and extensive information for assessment of fetal prognosis. The *EYA4* mutation detected by WGS in the present study, might also be related with the cardiac structural malformation, in addition to cadiomyopathy. Further researches should be performed to confirm this conclusion. We suggest that the WGS should be used to identify the underlying pathogenicity in cases with balanced translocation but abnormal clinical phenotypes. Balanced translocation could disrupt some critical genes and therefore individual development could be affected.

It is also possible that there might occur complex rearrangements involving three chromosomes of 5, 6 and 1. Due to the very small size of the breaking fracture in chromosome 1, it could not be detected by karyotyping. WGS did not detected possible breakpoint in chromosome 1 either. Another method such as metaphase FISH should be used to investigate the underlying fracture points. However, we could not perform the further investigation due to lack of sample.

In conclusion, we report a fetus with 1p36 deletion syndrome and a rare CHD phenotype of isolated dextrocardia. This is the first reported prenatal 1p36 deletion case presenting dextrocardia. Our report also indicates that genes in 1p36 are associated with not only cardiac structural anomalies, but also cardiac laterality development.

## Data Availability

All data were presented and available in main paper. Other phenotype or genotype information may also be obtained by contacting the corresponding author.

## Abbreviations

CMA: chromosomal microarray analysis
CHD: congenital heart disease
VSD: ventricle septal defect
BPD: biparietal diameter
HC: head curcumference
FL: femur length
WGS: whole genome sequencing
